# AGING AND CLIMATE CHANGE: FROM VULNERABILITY TO ACTION

**DOI:** 10.1093/geroni/igae098.0856

**Published:** 2024-12-31

**Authors:** Karl Pillemer

**Affiliations:** Chair

## Abstract

Despite the vulnerability of older people to climate change effects, as well as their potential to be part of climate change solutions, research has been limited. This multidisciplinary symposium responds to urgent calls for translational research on climate change as a critical pathway to reduce physical and mental health risks to older persons, brought about by climate-related hazards such as extreme temperature events, climate-related disasters, decreased air and water quality, forced migration, and vector-borne illnesses. Beyond vulnerability, panelists present research on older people’s actions to prevent and mitigate climate change. Two presentations provide new empirical findings on climate hazards. Bordes and Cherry examine how climate-related disasters affect health, using data from Hurricane Ida to examine differential effects on older and younger people. Koszalinski et al. examine effects of another climate-related hazard: harmful algae blooms, based on a longitudinal study of older adults in South Florida, demonstrating how such events can create harm from otherwise beneficial activities like outdoor nature activities. Two presenters focus on action taken by older people. Nikitin et al. address the intersection of the environment and attitudes toward aging, finding that nature contact is of special benefit to older persons. Liu and Pillemer examine intergenerational motivations for climate change activism among older people. Thiamwong’s presentation moves to community climate resilience strategies, presenting findings on community adaptation adapting to rising temperatures, with a focus on low-income older adults who are at special risk in extreme heat events.

